# Intussusception in a 20 weeks pregnant woman: a case report

**DOI:** 10.4076/1757-1626-2-6546

**Published:** 2009-08-05

**Authors:** Andreas Luhmann, Rachel Tait, Ahmed Hassn

**Affiliations:** Department of Surgery, Princes of Wales HospitalCoity Road, Bridgend, CF31 1RQUK

## Abstract

Intussusception in pregnancy is rare and poses unique diagnostic and therapeutic challenges. We present the case of a 22 year old, 20 weeks pregnant woman who presented with acute abdominal pain. Following abdominal ultrasound scanning she was diagnosed with intussusception. The patient underwent laparotomy and a small bowel resection was performed without any post operative complications. We review the literature to give a concise and up to date summary on the diagnosis and treatment of the condition with particular emphasis on the recently recognised usefulness of ultrasound scanning.

## Introduction

Intussusception is a rare but potentially life threatening (mother and child) complication in pregnancy challenging both gynaecologists and surgeons. Its diagnosis is difficult and emergency surgery indicated.

## Case presentation

**Figure 1.**
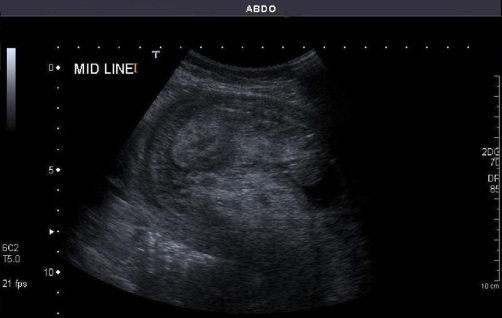
Ultrasound image showing features suggestive of intussusception.

A 20 weeks pregnant, 22-year-old nulliparous white woman of Polish origin was admitted through our accident and emergency department with acute onset of severe cramp like lower abdominal pain. Her pulse rate and blood pressure where normal. The initial abdominal examination was unremarkable and baseline blood tests, apart from a potassium of 2.9, where within normal limits. She was admitted by the gynaecological team for observation and an urgent pelvic ultrasound scan was performed. This did not show any pathology. The foetus was healthy. Later the same evening the patient was re-examined and a mobile mass was detected in the left upper quadrant of her abdomen. Our surgical team was contacted and we reviewed her the same night. At the time she was still in pain but had improved somewhat. Once again the mobile mass was palpable. Unsure of the diagnosis we organised an abdominal ultrasound scan for first thing in the morning. This showed features suggestive of intussusception (Figure 1).

Following discussion with the patient and the radiological and gynaecological consultants emergency surgery was performed. This showed a necrosed entero-enteric intussusception starting at the duodeno-jejunal flexure. We performed a small bowel resection with anastomosis of the small bowel to the 4th part of the duodenum. The total length of small bowel removed was 126 cm. Postoperative recovery was uneventful for mother and child and the patient was discharged 8 days after admission. There where no problems on follow up. Histology showed a benign polyp as leading lesion.

## Discussion

Intussusception is a rare but serious complication in pregnancy necessitating emergency surgery. Even though the patient is usually admitted under the care of the gynaecological team it is normally the surgeon who is asked to assist with the diagnosis and ultimately to operate. Intussusception is most common in childhood accounting for 15% of bowel obstructions [1]. Intussusception in adults accounts for only 5% of the total number of intussusceptions and only 1% of cases of bowel obstruction [2]. Its incidence is 1/30000 hospital admissions or 1/3000 of all abdominal operations [3]. The mean age at presentation for adults is between 51 - 54.4 years [3,4]. The male to female ratio is 1:1-1.3 [3]. Symptoms on presentation are most often secondary to obstruction and include nausea, vomiting, constipation, abdominal pain and a mass [2]. Contrary to childhood intussusception a leading lesion is found in up to 95% of cases [1-4]. These can be of many different aetiologies including carcinoma of the colon, meckel’s diverticulum, submucous lipomas, Peutz Jegher polyps, leiomyomas, neurofibromas, adenomas, heterotopic pancreatic tissue and many others [5,6]. The literature differs between small bowel [3] and ileo-colic [7] as the most common types of intussusception. They agree that small bowel intussusception is more likely to have a benign lesion at the apex whereas ileo-colic or colo-colic are more likely to have malignant ones [2,3,7].The imaging modalities of choice in adults are the CT/ MRI scan and the double contrast enema [1,2]. Their diagnostic yields are 52%-58% and 41%-73%, respectively. The abdominal X-ray usually shows features of obstruction. The classical finding on CT is the “target lesion” formed by one part of the bowel telescoped into another [8].

The treatment of intussusception in the adult is almost always surgery [1-4]. Intussusception is difficult to diagnose at the best of times but in pregnancy even more so. Some of the presenting symptoms like abdominal pain and vomiting are common symptoms in pregnancy. Furthermore, it is not only the life of the mother that is at risk but also the life of the foetus.

Intussusception in pregnancy is rare. Intestinal obstruction in pregnancy has been reported to occur with an incidence between 1:2500-3500, most commonly secondary to adhesions or gastrointestinal volvolus [6]. Intestinal obstruction in pregnancy is associated with a high maternal and perinatal mortality of 6% and 26%, respectively [9]. Additionally, diagnostic imaging in pregnancy is restricted to USS and MRI, with MRI not readily available in many hospitals. In our case the diagnosis was made by abdominal ultrasound scan. Recent publications have shown ultrasound scan to be a useful imaging technique in intussusception in pregnancy [7,10]. It can exclude many of the differential diagnoses such as a twisted ovarian cyst and does not carry the risk of radiation to the foetus.

## Conclusions

Even though it is a rare occurrence, surgeons should be aware of the possibility of intussusception in pregnancy - it needs urgent and decisive intervention. Ultrasound scanning is a safe investigation in pregnancy and frequently confirms the diagnosis.

## Abbreviations

CT: computer tomography
MRI: magnet resonance imaging
USS: ultrasound scan
